# Mouse fitness measures reveal incomplete functional redundancy of Hox paralogous group 1 proteins

**DOI:** 10.1371/journal.pone.0174975

**Published:** 2017-04-05

**Authors:** James S. Ruff, Raed B. Saffarini, Leda L. Ramoz, Linda C. Morrison, Shambralyn Baker, Sean M. Laverty, Petr Tvrdik, Mario R. Capecchi, Wayne K. Potts

**Affiliations:** 1 Department of Biology, University of Utah, Salt Lake City, Utah, United States of America; 2 Department of Mathematics and Statistics, University of Central Oklahoma, Edmond, Oklahoma, United States of America; 3 Department of Neurosurgery, University of Utah, Salt Lake City, Utah, United States of America; 4 Department of Human Genetics, University of Utah, Salt Lake City, UT, United States of America; University of Iceland, ICELAND

## Abstract

Here we assess the fitness consequences of the replacement of the *Hoxa1* coding region with its paralog *Hoxb1* in mice *(Mus musculus)* residing in semi-natural enclosures. Previously, this *Hoxa1*^*B1*^ swap was reported as resulting in no discernible embryonic or physiological phenotype (i.e., functionally redundant), despite the 51% amino acid sequence differences between these two Hox proteins. Within heterozygous breeding cages no differences in litter size nor deviations from Mendelian genotypic expectations were observed in the outbred progeny; however, within semi-natural population enclosures mice homozygous for the Hoxa1^B1^ swap were out-reproduced by controls resulting in the mutant allele being only 87.5% as frequent as the control in offspring born within enclosures. Specifically, Hoxa1^B1^ founders produced only 77.9% as many offspring relative to controls, as measured by homozygous pups, and a 22.1% deficiency of heterozygous offspring was also observed. These data suggest that *Hoxa1* and *Hoxb1* have diverged in function through either sub- or neo-functionalization and that the HoxA1 and HoxB1 proteins are not mutually interchangeable when expressed from the *Hoxa1* locus. The fitness assays conducted under naturalistic conditions in this study have provided an ultimate-level assessment of the postulated equivalence of competing alleles. Characterization of these differences has provided greater understanding of the forces shaping the maintenance and diversifications of Hox genes as well as other paralogous genes. This fitness assay approach can be applied to any genetic manipulation and often provides the most sensitive way to detect functional differences.

## Introduction

Approximately 10% of genes show minimal-to-no phenotypic consequences when disrupted in mice (*Mus musculus*) [1]. A common explanation is functional redundancy, which argues that genes code for overlapping functions [2,3]. Often, studies concluding functional redundancy are assessed using only proximate measures (e.g., biomarkers, gene expression, and histology) in artificial environments, where ecological pressures shaping the evolutionary history of the genes of interest and the organisms that harbor them are largely absent. A naturalistic competitive environment and the incorporation of ultimate measures (i.e., fitness measures) could be useful in differentiating between complete and partial redundancy of disrupted genes.

Much empirical and theoretical effort has been applied to understanding how duplicated genes can be maintained through evolutionary time. When initially duplicated, one copy is likely to accumulate degenerative mutations (in either protein coding or regulatory sequences), which are invisible to selection, leading to non-functionalization [4]. Despite this mutational onslaught numerous duplications have been maintained, and gene families are commonplace, within genomes across taxa. Alternative paradigms for the fate of duplicated genes include neo-functionalization, in which mutations in the protein or regulatory sequences of a duplicated gene lead to a novel function, and sub-functionalization, wherein ancestral gene functions are partitioned (reviewed in [5,6]). Importantly, the processes of neo- and sub-functionalization should not result in complete functional redundancy, but instead in overlapping or incomplete redundancy inversely proportional to the degree of functional divergence or partitioning.

Originally discovered in *Drosophila*, *Hox* genes encode developmentally important transcription factors that provide insightful clues to mechanisms of gene and genome duplications during the evolution of metazoans. In mice and humans, there are 39 Hox genes, arranged in four tightly linked clusters (HoxA, HoxB, HoxC, and HoxD) on four different chromosomes, each harboring 9–11 genes belonging to 13 different paralogous groups (Hox1-Hox13). The classic view is that these four Hox clusters have derived from a single early chordate cluster in two rounds of whole genome doubling [7], although advanced phylogenetic techniques favor the hypothesis that these blocks of Hox paralogy have resulted from small-scale events, including segmental duplications, independent gene duplications and translocations [8].

Molecular properties of Hox genes from different paralogous groups have diverged significantly. In addition to displaying differential temporal and anterior-posterior expression patterns, different Hox paralogs recruit distinct DNA binding co-factors. Moreover, their DNA binding domains, the homeoboxes, are not functionally interchangeable in the development of most tissues, as demonstrated in elegant homeobox-swapping experiments by Zhao and Potter [9,10]. These findings are consistent with the hypothesis that the primordial Hox cluster was formed by ancient gene duplication events [11]. However, within a given paralogous group, the Hox transcription factors remain remarkably conserved. High degree of functional equivalence has been shown by transgenic approaches as well as by precise gene replacements in Hox paralogous groups 11, 3, and 1 [12–14]. The latter study found that two Hox1 paralogs, *Hoxa1* and *Hoxb1*, both of which are involved in the patterning of the brainstem, are functionally interchangeable at the protein level (via proximate assessments).

In *Hoxa1* knockout mice, expression of *Hoxb1* and other downstream genes is altered, the brainstem respiratory circuits are malformed, and *Hoxa1* neonatal knockouts die [14,15]. Conversely, *Hoxb1* knockouts are viable, but display facial paralysis [16]. Intriguingly, relative to wild type, homozygous *Hoxa1*^*B1*^ swapped mice, mutants expressing HoxB1 protein from both *Hoxa1* alleles, show no changes under laboratory conditions; this is despite a 15% amino acid sequence divergence at the homeodomains and merely 49% total identity [14,17]. Thus, the laboratory phenotypic assessment suggested that if expressed at sufficient levels, either protein could execute the program carried out by the other. This finding is further supported by additional work in *Xenopu*s, in which functional redundancy across Hox paralogous group 1 was demonstrated in a variety of proximate measures [18].

In a previous experiment, we assessed the fitness consequences of mice being homozygous for a *Hoxb1*^*A1*^ swap compared to controls using our organismal performance assay (OPA) model system [19]. OPAs are a type of fitness assay in which house mice compete among each other for limited resources in a semi-natural environment. Treatment and control individuals compete directly, and the performance of individuals is measured in terms of Darwinian fitness (i.e., reproductive success); this approach has been used previously to quantify the adverse effects of numerous genetic, nutritional, and pharmaceutical manipulations [20–26]. Specifically, in the case of the *Hoxb1*^*A1*^ swap, we observed that males homozygous for the swap acquired fewer territories, a key fitness component, and that a deficiency of both *Hoxb1*^*A1*^ homozygous and heterozygous offspring was present in the generation of offspring born within OPA enclosures, despite detailed molecular studies indicating functional interchangeability. These findings suggest that *Hoxa1* and *Hoxb1* are phenotypically divergent, and consequently, have incomplete redundancy at the organismal level.

Due to the surprising nature of our initial discovery on incomplete redundancy of Hox paralogous group 1 proteins we set out to find independent, complementary supporting evidence. Here we use OPAs to assess the fitness of the reciprocal swap—mice expressing HoxB1 protein from the *Hoxa1* locus (*Hoxa1*^*B1*^*)—*relative to control mice bearing an appropriate genetic control. During these 25-week OPA trials we take measures of male competitive ability of founding individuals and assess allelic frequencies and *Hoxa1* genotypes of offspring born within enclosures for numerical deficiencies or excesses. Additionally, breeding cage measurements of litter size and genotypic frequencies of offspring from heterozygous mates are taken, allowing us to evaluate the role of naturalistic environments in revealing fitness differentials. Such assessments of reciprocal swaps are common in developmental literature (e.g., [13,14,27]), and of particular importance when the severity of the respective knockouts differs, as it does for these paralogs [14–16].

## Materials and methods

### Animals

**Fig 1:**
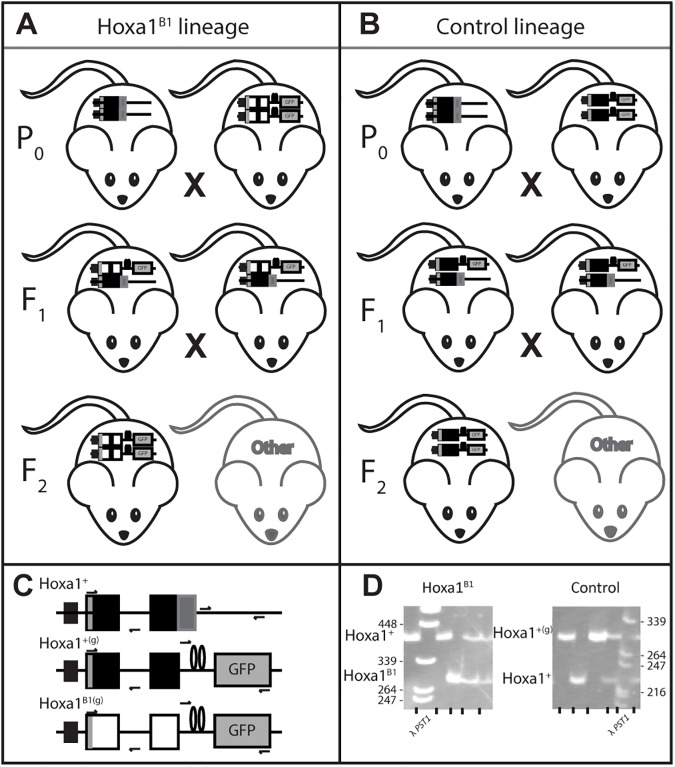
Breeding design of Hoxa1^B1^ and control founders. (A) To produce animals with natural behavior bearing *Hoxa1*^*B1*^swaps, *Hoxa1*^*B1(g)/B1(g)*^ 129 x C57BL/6 mice were mated to mice from a wild-derived colony. Heterozygotes were then crossed and confirmed *Hoxa1*^*B1(g)/B1(g)*^ progeny were selected as founders. (B) Control animals were bred by crossing *Hoxa1*^*+(g)/+(g)*^ 129 x C57BL/6 mice with the same wild-derived colony. The *Hoxa1*^*+(g)/+*^ were then crossed to produce the F_2_ generation. Again, genotypes were confirmed and *Hoxb1*^*+(g)/+(g)*^ mice were selected as founders. (C) Cartoons of wild type (*Hoxa1*^*+*^), wild type with the IRES-tauGFP tag (*Hoxa1*^*+(g)*^), and *Hoxa1*^*B1*^ swap with the IRES-tauGFP tag (*Hoxa1*^*B1(g)*^) are provided. Large rectangles represent exons one and two of *Hoxa1* (black) and *Hoxb1* (white). The *Hoxa1* promoter is conserved across all genotypes. Loops separating the tauGFP tag from the second exon depict the internal ribosomal entry site. Primer binding sites are shown by arrows. (D) Photograph of polyacrylamide gel discrimination between *Hoxa1*^*B1(g*)^, and *Hoxa1*^*+*^ (left) and between *Hoxa1*^*+(g)*^ and *Hoxa1*^*+*^(right) alleles.

Strains of inbred laboratory mice do not possess the natural behaviors required for OPA assessment [28,29]; therefore, suitable mice with the *Hoxa1*^*B1*^ swap and an appropriate control were bred (Fig 1). A Hoxa1^B1^ treatment lineage was bred by crossing 16 *Hoxa1*^*B1(g)/B1(g)*^–possessing 129x C57BL/6 hybrids, generated by homologous recombination (see reference [14]), with genetically diverse wild-derived mice from the 8^th^ generation of the colony originally described by Meagher et al. [20]. The resulting heterozygotes (F_1_) were crossed (n = 58) to establish the F_2_ generation (Fig 1A). Only confirmed *Hoxa1*^*B1(g)/B1(g)*^ homozygotes, here on referred to as Hoxa1^B1^ founders, were selected for OPA assessment. Likewise, a control lineage of mice was bred by crossing 17 *Hoxa1*^*+(g)/+(g)*^ 129 x C57BL/6 hybrid mice with the same wild-derived stock. The resulting F_1_ *Hoxa1*^*+(g)/+*^ generation (n = 85) were then crossed to produce the F_2_ generation (Fig 1B). Only confirmed *Hoxa1*^*+(g)/+(g)*^ homozygotes, here on referred to as control founders, were selected as OPA founders. Prior to OPA release, all animals were housed according to standard protocols under a 12:12 h light:dark cycle with food and water available *ad libitum*. All protocols were approved by the animal care guidelines of the IACUC at the University of Utah (Protocol Numbers 02–09017, 05–08012).

### Genotyping

*Hoxa1* genotype was determined using a three primer PCR amplification system where a 3' common primer (5'- TCA CCA TCA CCA CCA TCA C) anneals between exons 1 and 2 within the bridging intron and specific 5' primers, which anneal within exon 1—for Hoxa1^+/+^ (5'-ACT CCT TAT CCC CTC TCC AC) and for Hoxa1^B1(g)/B1(g)^ (5'-CAA GAG AAA CCC ACC TAA GAC)—yield a 381 bp and 284 bp fragment respectively (Fig 1C). These amplicons were visualized on 5% polyacrylamide gels (Fig 1D). Likewise, to distinguish between tauGFP-tagged wild type and true wild type individuals a similar genotyping system was used. A 3' common primer (5'-AGA TGG GAC GAG AAA GGA AG) and a 5' specific primer for Hoxa1^+(g)/+(g)^ (5'-ACA ACC ACT ACC TGA GCA A) located within the tauGFP site and for Hoxa1^+/+^ (5'- TGG CAG CGA TGA GAA AAC), located 5' of where the tauGFP insertion within animals possessing it, yield a 298 bp and 225 bp fragment respectively, which were visualized on 5% polyacrylamide gels (Fig 1D). This approach was used to acquire genotypes of 95.3% of F_2_ progeny, and 97.4% of F_3_ pups from OPA enclosures.

### OPA enclosures

OPA enclosures are 30 m^2^ and subdivided into six subsections by wire mesh to encourage territorial formation. Subsections contain *ad libitum* food and water dispensers associated with a set of nest boxes in one of the four “optimal” territories (with dark enclosed nest boxes) or two “suboptimal” territories (with nest boxes exposed to the light). Descriptions of enclosure, including photographs and associated methods, have been provided elsewhere in more detail [19,23–25].

Three independent OPA enclosures were founded by 30 F_2_ individuals, 10 males and 20 females, with equal numbers of Hoxa1^B1^ and control founders within each population. Founders were 38.4 ± 0.9 (mean ± SD) weeks of age for females and 37.8 ± 0.3 weeks for males. Relatedness was avoided within populations and no individuals were related at or above the sibling level. To prevent incidental breeding before the establishment of social dominance networks by males, untreated females from our stock wild-derived colony were initially released with male founders allowing territorial formation prior to release of female founders. At the start of the 25-week OPA trial untreated females were removed and female founders released.

Population-level reproductive success was determined for founders of each treatment by harvesting tissue samples gathered during “pup sweeps”, in which pups born during the previous cycle were removed from the population and euthanized. Sweeps occurred every five weeks to prevent offspring born in enclosures from becoming breeders.

Prior to release into enclosures, founders of both sexes were implanted with unique passive integrated transponder tags (TX1400ST, BioMark, Boise ID). Location data are gathered via a set of antennae and readers (FS2001F-ISO, BioMark, Boise ID) placed at each of the feeders and data are recorded with data-logging software (Minimon, Culver City, CA). Dominance was assigned when a male had more than 80% of all male readings at a single feeder over the course of a multi-day reader session. Paired measures of the number of territories controlled by Hoxa1^B1^ and control males were gathered for each population throughout the study.

### Statistical methods

Deviations from expected genotypic frequencies of F_2_ offspring were assessed in Hoxa1^A1^ treatment and control lineage heterozygote breeding cages. Each homozygote genotype was directly compared to the other and heterozygote counts were compared to the summation of both homozygote counts. A generalized linear mixed model (GLMM) with a Poisson distribution and a logarithmic link was used, in which genotype was modeled as a fixed effect and individual breeding cage was modeled as a random effect. Hoxa1^B1^ treatment lineage models were based on 116 observations from 58 breeding cages, while both models for the control lineage were based on 170 observations from 85 cages. Litter sizes between the lineages were compared with a Mann-Whitney U test.

To examine the counts of the mutant *Hoxa1*^*B1(g)*^ and control *Hoxa1*^*+(g)*^ alleles of offspring born within OPAs a GLMM assuming a Poisson distributions and using a logarithmic link was employed. We predicted population-level allelic counts by modeling the fixed effects of allele, time, and their interaction, while population was modeled as a random effect with a generated random intercept and slope. Allelic counts were measured five times, at five-week intervals, for a total of 30 observations across the three populations and the intercept was set at the grand mean (week 15).

Similar to the analysis of genotypes from breeding cages, offspring counts from OPAs were modeled over time using two GLMMs with Poisson distributions and logarithmic links. In the first model, we predicted population-level fitness by modeling the fixed effects of genotype (*Hoxa1*^*B1(g)/B1(g)*^ *vs*. *Hoxa1*^*+(g)/+(g)*^), time, and their interaction, while population was modeled as a random effect with a generated random intercept. Offspring genotypes were measured five times, at five-week intervals, for a total of 30 observations across the three populations and the intercept was set at the grand mean (week 15). In the second model, four genotype groups (observed heterozygotes, summed homozygotes, and the expected (2X) count of heterozygotes based upon each of the homozygote counts) were assessed with 15 observations available for each. Time, genotype, and their interaction were modeled as fixed effects and population was modeled as a random effect with a random intercept and slope.

As the six territories per population can only be occupied or not, we used a GLMM with a binomial distribution and a logit link to model the fixed effects of genotype, time, and a time by genotype interaction on territorial control (occupied territories versus unoccupied), with population modeled as a random effect. Territorial occupation was assessed across the study for a total of 70 observations. The intercept of the model was set at the grand mean (week 12.8).

All GLMMs were fit in R using the *glmer* function of the ‘lme4’ library [30,31]. For each GLMM, multiple candidates for the random effects terms were generated, including models estimating both intercept and/or slope for random effects—in all cases the model that had the lowest Akaike information criterion (AIC) score was selected.

## Results

Genotypic frequencies of F_2_ offspring produced within both Hoxa1^B1^ and control lineage heterozygous breeding cages conformed to Mendelian expectations and litters from both lineages were of similar size (Table 1). In Hoxa1^B1^ lineage heterozygous breeding cages (n = 58), no difference was observed in the count of homozygous offspring possessing the two genotypes (GLMM; Z = 1.54, *p* = 0.123; S1 Table); likewise, neither a deficiency nor excess, of heterozygotes was observed (GLMM, Z = -0.43, *p* = 0.665). Similarly, in control lineage heterozygous breeding cages (n = 85) no differences were observed between the two homozygote counts (GLMM, Z = -0.386, *p* = 0.700), nor did the count of heterozygotes differ from that of summed homozygotes (GLMM, Z = 1.08, *p* = 0.283). Finally, F_2_ litters did not differ in size between Hoxa1^B1^ and control lineages (Mann-Whitney, U = 2452, *p* = 0.958).

**Table 1:** Summary of genotypic counts and litter sizes of Hoxa1^B1^ treatment and control lineage breeding cages.

Lineage (N)	Mutant Homozygotes	Wild type Homozygotes	Heterozygotes	TOTAL
Hoxa1^B1^ (58)	1.7 ± 0.2^*a*^	2.1 ± 0.2	3.7 ± 0.2	**7.5 ± 0.2**
Control (85)	2.0 ± 0.1	1.9 ± 0.1	3.6 ± 0.2	**7.4 ± 0.2**

^*a*^ Values are means ± SEs.

**Fig 2:**
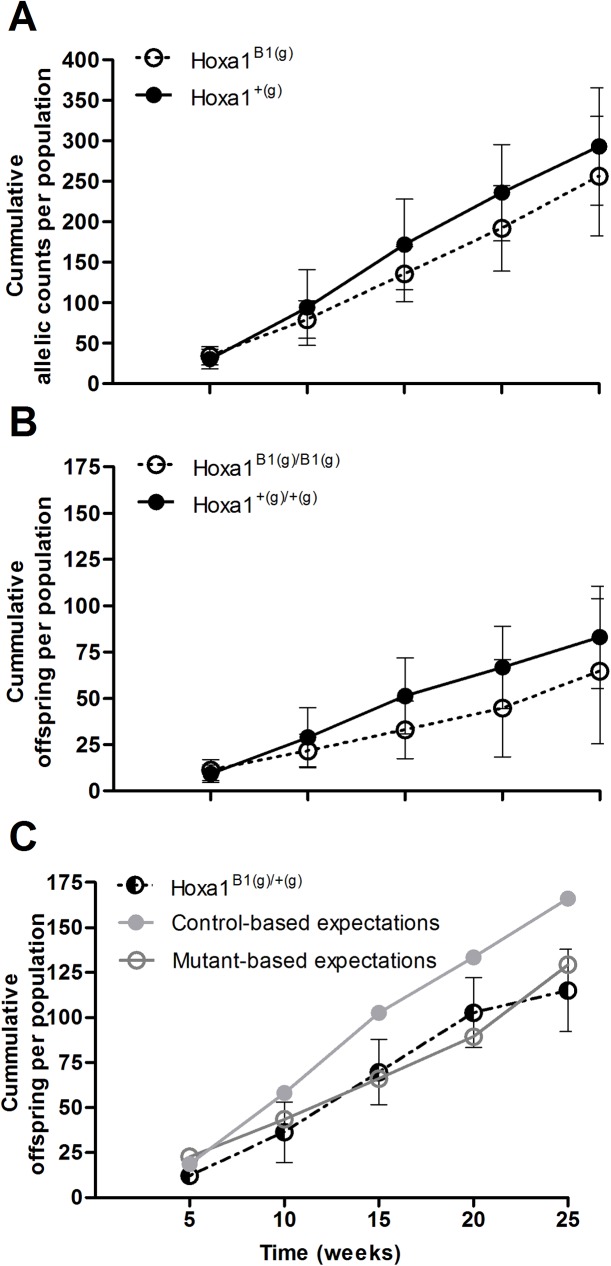
**Cumulative allelic (A) homozygote (B) and heterozygote (C) counts of offspring born in OPAs. (A)** The mutant *Hoxa1*^*B1(g*)^ allele was only 87.5% as frequent as the control allele within offspring (GLMM; Z = 2.89, *p* = 0.004. (B) Hoxa1^B1^ founders produced only 77.9% as many offspring relative to controls as measured by homozygous pups (GLMM; Z = 2.75, *p* = 0.006). (C) Similarly, a 22.1% deficiency of heterozygous offspring was detected relative to summed homozygotes (GLMM; Z = 2.24, *p* = 0.025). Heterozygote levels were below those predicted based on the count of *Hoxa1*^*+(g)/+(g)*^ pups (GLMM; Z = 4.09, *p* < 0.001; Gray line with filled circles), but did not differ from predictions based on *Hoxa1*^*B1(g)/B1(g)*^ offspring (Gray line with open circles). All genotypes were assessed at multiple time points across populations (*n* = 3) for a total of 15 observations. Black lines connect population means and error bars represent standard error.

Within offspring born in OPA enclosures the mutant *Hoxa1*^*B1(g*)^ allele was only 87.5% as common as the control allele (Fig 2A). Specifically, there were 48.7 (+5.3, -4.8; mean ± SE) mutant *Hoxa1*^*B1(g*)^ alleles per population per sampling, while for the control *Hoxa1*^*+(g)*^ allele there were 56.3 (+2.9, -2.7); this difference was statistically significant (GLMM; Z = 2.89, *p* = 0.004; S2 Table). Additionally, there was a significant increase in allelic counts over the course of the study (GLMM; Z = 1.99, *p* = 0.046), but this increase did not differ between the two alleles (GLMM; Z = -1.38, *p* = 0.168). SEs are asymmetrical, as they are back transformed from logarithmic data.

As measured by homozygous offspring, Hoxa1^B1^ founders produced only 77.9% as many pups as controls over the course of the study within OPAs (Fig 2B). Specifically, there were 12.2 (+1.0, -0.9) *Hoxa1*^*B1(g)/B1(g)*^ offspring per sampling per population, while there were 16.0 (+ 1.6, -1.5) *Hoxa1*^*+(g)/+(g)*^ offspring; this difference was statistically significant (GLMM; Z = 2.75, *p* = 0.006; S2 Table). Neither an effect of time (GLMM; Z = 1.18, *p* = 0.238) nor a time-by-genotype interaction (GLMM; Z = -1.26, *p* = 0.207) influenced offspring counts, suggesting a consistent rate of reproduction across the study.

Relative to summed counts of homozygotes, a 22.1% deficiency of heterozygous offspring was detected over the course of the study within OPAs (Fig 2C). Specifically, there were 24.1 (+2.7, -2.1) *Hoxa1*^*B1(g)/+(g)*^ heterozygotes per sampling per population, while there were 28.3 (+2.1, -2.0) homozygotes; this difference was statistically significant (GLMM; Z = 2.24, *p* = 0.025; S2 Table). Consistent with the homozygous comparison, no effect of time (GLMM; Z = 1.43, *p* = 0.152) nor time-by-genotype (GLMM; Z = -0.71, *p* = 0.475) were detected on genotypic counts. Heterozygote levels were below those predicted based on the count of *Hoxa1*^*+(g)/+(g)*^ pups (GLMM; Z = 4.09, *p* < 0.001), but did not differ from predictions based on *Hoxa1*^*B1(g)/B1(g)*^ offspring (GLMM; Z = 0.20, *p* = 0.843).

**Fig 3:**
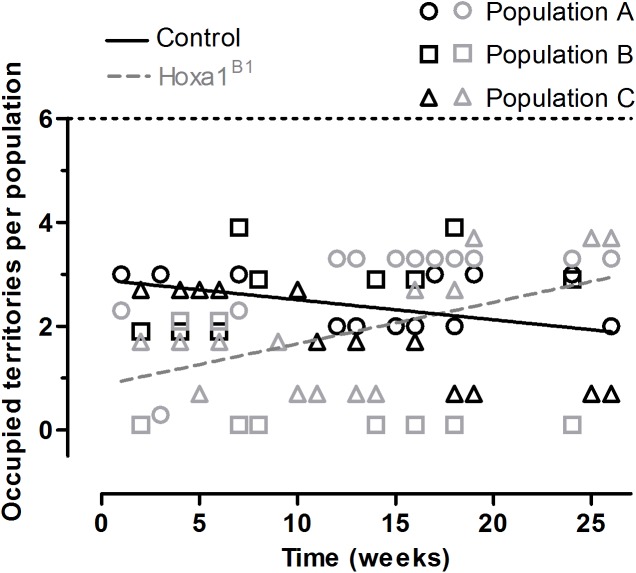
Competitive ability of Hoxa1^B1^ and control founders in OPAs. No consistent relationship between genotype and competitive ability was observed. Initially, fewer territories were occupied by Hoxa1^B1^ founders than controls leading to a marginally significant difference at the grand mean (GLMM; Z = 1.96, *p* = 0.050); however, this pattern degraded over time (GLMM; Z = -3.12, *p* < 0.002) and reversed by the end of the study. Territorial occupation of both groups was assessed at multiple time points across populations (*n* = 3) for a total of 70 observations. Provided lines are simple linear regressions based upon raw data to help illustrate overall significantly differing slopes over time between treatments. Populations are identified by shape and paired at each timepoint (black outline for control and gray outline for Hoxa1^B1^ founders) and cannot sum to more than six (dashed horizontal line) as this is the maximum number of territories per population.

Within OPAs, no uniform difference in territorial ownership across the study was observed between male Hoxa1^B1^ and control founders (Fig 3). Initially, Hoxa1^B1^ founders obtained fewer territories than controls leading to a marginally significant difference at the model intercept (GLMM; Z = 1.96, *p* = 0.050; S3 Table). Specifically, at week 12.8 the probability that one of the six territories per population was controlled by a male Hoxa1^B1^ founder was 0.30, while for control founders the probability was 0.40 (leaving a 0.3 probability that a territory was unoccupied); however, the probability that a Hoxa1^B1^ male controlled a territory increased over time relative to controls (GLMM; Z = -3.12, *p* = 0.002) leading to a situation in which the pattern observed initially was reversed by the study’s conclusion. Finally, there was no differential longevity between Hoxa1^B1^ and control founders, as all founders (n = 90) survived until the end of the 25-week study.

## Discussion

Hoxa1^B1^ founders were outcompeted by controls within OPA enclosures as they produced only 77.9% as many offspring relative to controls as measured by homozygous pups; furthermore, a 22.1% deficiency of heterozygous offspring was observed in enclosures. Combined, the homozygote and heterozygote deficiencies resulted in the mutant *Hoxa1*^*B1(g*)^ being only 87.5% as frequent as the control allele in offspring born within OPAs. These OPA observations are in contrast to measures in standard laboratory breeding cages where neither a difference in litter size nor genotypic frequencies were observed between heterozygous pairs in the Hoxa1^B1^ and control lineage. Typically within OPAs differential reproductive output between experimental groups is driven by either differential longevity or male competitive ability; however, neither of these fitness components differed between Hoxa1^B1^ and control founders. Though an overall trend emerged that male control mice were more likely to dominate a territory than Hoxa1^B1^ males, the instability of the pattern over time makes it difficult to link competitive ability to the observed reproductive deficiency. Furthermore, as all founders survived until the termination of the study, differential reproduction cannot be explained by mortality.

The reproductive differential between Hoxa1^B1^ and control founders, in the broadest sense, could be due to either litters containing fewer pups or decreased frequency of litter production. As Hox genes have key roles in development it is possible that embryonic lethality could be reducing the size of litters born to Hoxa1^B1^ founders, though this is not supported by cage measures of genotypic frequency. Hoxa1B1 founders could be having fewer litters throughout the study due to a variety of factors such as decreased resource acquisition, or discrimination during mate choice; however, no evidence is available to specifically support that there are fewer litters produced. Thus, the proximate underpinnings of the fitness differential identified here require further investigation.

The results of this study are consistent with our previous investigation using OPAs to assess the fitness consequences of the reciprocal *Hoxb1*^*A1*^ swap [19]. In that study we illustrated that mice homozygous for the swap had only 64.4% as many offspring as controls, and likewise detected a 16.2% deficiency in heterozygous offspring born within enclosures; the direction and magnitude of these reproductive deficiencies is similar to that observed in this report. Conversely, the previous investigation also detected that males homozygous for the swap acquired and held territories at a lower rate than did controls, a finding not corroborated in this investigation; this is possibly due to differential effects of the respective reciprocal swaps. Collectively, both OPA studies support incomplete redundancy for HoxA1 and HoxB1 proteins with OPA fitness measures. In both studies, distorted gene segregations in OPAs were determined after normal frequencies of gene swap alleles were seen in the offspring from outbred heterozygous breeders maintained in standard laboratory cages. Thus, a naturalistic competitive environment reveals incomplete redundancy even in the robust wild type genetic background, which masks reproductive deficits in the laboratory breeding conditions.

In regards to genotypic frequencies observed in heterozygous breeding cages, neither study found deviations from Mendelian expectations for either the experimental or control lineages generated in this research. In the original report, Tvrdik and Capecchi [14] observed a decreased segregation of the *Hoxa1*^B1(g)/B1(g)^ genotype in the mixed inbred C57BL/6 x 129 background kept in laboratory conditions. The distortion of *Hoxa1*^B1(g)/B1(g)^ segregation was noticeable, although it did not reach statistical significance. We argue that this discrepancy is due to the pivotal role of *Hoxa1* in the hindbrain development, being positioned in the center of a complex regulatory network of upstream and downstream factors that can modify the severity of the mutant or hypomorphic phenotype [32–35].

The strong influence of genetic background on *Hoxa1* function is corroborated by the heterogeneity of human syndromes involving *HOXA1* mutations. Homozygous *HOXA1* loss of function was found in consanguineous families from three ethnically unrelated populations of Saudi Arabian, Turkish, and Native American descent. These *HOXA1* mutations result in two overlapping, but distinct syndromes which have been described as the Bosley-Salih-Alorainy syndrome and the Athabaskan brainstem dysgenesis syndrome. The differences in manifestation of the two syndromes are most likely attributable to ethnic-specific genetic modifiers present in these human populations [36–39].

Two previous studies supporting functional redundancy of Hox paralogous group 1 proteins have been conducted which carefully assessed multiple proximate endpoints including expression levels and histological confirmation of normal tissue throughout development [14,18]. The degree to which Hox1 paralogs are able to compensate for the under-expression, or complete removal, of a paralog is astounding; however, complete functional redundancy can only be assessed at the ultimate (fitness) level. Therefore, our experiments should not be seen as antagonistic to these previous studies, but as an evolutionarily compliment capable of revealing the cryptic phenotypes associated with these, or other manipulations.

*Hox* genes have been studied intensely to further our understanding of how duplicated genes are maintained across evolutionary time, with the leading explanation for the case of *Hox* paralogs being through the process of sub-functionalization [5]. Importantly, previous reports of functional redundancy between paralogous genes have been cited as evidence for this model, though we argue that if sub-functionalization has occurred, then incomplete redundancy (as opposed to complete) should be observed, indicating that partitioning, and possible expansion, of ancestral gene function has occurred to some degree. As the degree of partitioning increases over evolutionary time, the level of redundancy should proportionally decrease. What is needed to assess the degree to which gene function has changed between paralogs are assessments at both proximate and ultimate levels to better quantify the degree to which paralogs are functionally redundant.

Beyond inquiries concerning the maintenance of duplicated genes, many questions in both genetics and evolutionary biology require experimentally derived fitness measures. Specifically, within areas of research including facultative sex ratio biasing, Hamilton-Zuk sexual selection, and the benefits of genome-wide heterozygosity, the need for experimentally derived fitness measures has already been acknowledged [40–42]. Though such systems have not been readily adopted by those working with vertebrates, fitness assays using yeast and invertebrates have proven invaluable in unraveling other great evolutionary puzzles including quantifying the costs of mutation accumulation, the benefits of sexual reproduction, and the function of ‘nonessential’ genes [43–45].

## Conclusion

Using the OPA model system we are able to quantify negative fitness consequences in mice homozygous for a *Hoxa1*^*B1*^ swap relative to controls, adversity that was not observed previously by studies focusing solely on proximate endpoints. With OPAs, we and others have been able to detect adversity to numerous exposures (e.g., added sugar diets and pharmaceuticals) and genetic manipulations (e.g., cousin- and sibling-level inbreeding, and harboring the *t*-Complex) that were missed by other, often molecular, assays [20–26]. Successful examples of fitness assays, such as OPAs, illustrating cryptic phenotypes from genetic manipulations support their use in functional genomics, especially when confronted with disruptions that lead to “no-phenotype” relative to controls [46]. The illustration that HoxA1 and HoxB1 protein do not fully recapitulate each other’s phenotypes, suggests that these paralogs have diverged in gene function over time, likely through sub-functionalization.

## Supporting information

S1 Table Summary of mixed model results for genotype frequencies in Hoxa1^B1^ treatment and control lineage heterozygous F_1_ breeding cages. (PDF)

S2 Table Summary of mixed model results for allelic and genotypic counts of offspring born within OPAs. (PDF)

S3 Table Summary of mixed model results for founder competitive ability within OPAs. (PDF)

## References

[1] Barbaric I, Miller G, Dear TN. Appearances can be deceiving: Phenotypes of knockout mice. Briefings Funct Genomics Proteomics. 2007;6: 91–103. 10.1093/bfgp/elm008 17584761

[2] Nowak MA, Boerlijst MC, Cooke J, Maynard Smith J. Evolution of genetic redundancy. Nature. 1997;388: 167–71. 10.1038/40618 9217155

[3] Kafri R, Springer M, Pilpel Y. Genetic Redundancy: New Tricks for Old Genes. Cell. 2009;136: 389–392. 10.1016/j.cell.2009.01.027 19203571

[4] Ohno S. Evolution by gene duplication. New York: Springer; 1970.

[5] Prince VE, Pickett FB. Splitting pairs: the diverging fates of duplicated genes. Nat Rev Genet. 2002;3: 827–837. 10.1038/nrg928 12415313

[6] Innan H, Kondrashov F. The evolution of gene duplications: classifying and distinguishing between models. Nat Rev Genet. 2010;11: 97–108. 10.1038/nrg2689 20051986

[7] Furlong RF, Holland PWH. Were vertebrates octoploid? Philos Trans R Soc Lond B Biol Sci. 2002;357: 531–544. 10.1098/rstb.2001.1035 12028790 PMC1692965

[8] Abbasi AA. Diversification of four human HOX gene clusters by step-wise evolution rather than ancient whole-genome duplications. Dev Genes Evol. 2015;225: 353–357. 10.1007/s00427-015-0518-z 26481129

[9] Zhao Y, Potter SS. Functional specificity of the Hoxa13 homeobox. Development. 2001;128: 3197–3207. 11688568 10.1242/dev.128.16.3197

[10] Zhao Y, Potter SS. Functional comparison of the Hoxa 4, Hoxa 10, and Hoxa 11 homeoboxes. Dev Biol. 2002;244: 21–36. 10.1006/dbio.2002.0595 11900456

[11] Garcia-Fernàndez J. Hox, ParaHox, ProtoHox: facts and guesses. Heredity;2005;94: 145–152. 10.1038/sj.hdy.6800621 15578045

[12] Zákány J, Gérard M, Favier B, Potter SS, Duboule D. Functional equivalence and rescue among group 11 Hox gene products in vertebral patterning. Dev Biol. 1996;176: 325–328. 10.1006/dbio.1996.0137 8660870

[13] Greer JM, Puetz J, Thomas KR, Capecchi MR. Maintenance of functional equivalence during paralogous Hox gene evolution. Nature. 2000;403: 661–665. 10.1038/35001077 10688203

[14] Tvrdik P, Capecchi MR. Reversal of Hox1 gene subfunctionalization in the mouse. Dev Cell. 2006;11: 239–50. 10.1016/j.devcel.2006.06.016 16890163

[15] del Toro ED, Borday V, Davenne M, Neun R, Rijli FM, Champagnat J. Generation of a novel functional neuronal circuit in Hoxa1 mutant mice. J Neurosci. 2001;21: 5637–5642. 11466434 10.1523/JNEUROSCI.21-15-05637.2001 PMC6762659

[16] Goddard JM, Rossel M, Manley NR, Capecchi MR. Mice with targeted disruption of Hoxb-1 fail to form the motor nucleus of the VIIth nerve. Development. 1996;122: 3217–3228. 8898234 10.1242/dev.122.10.3217

[17] Remacle S, Abbas L, Backer O De, Gavalas A, Gofflot F, Jacques J, et al Loss of Function but No Gain of Function Caused by Amino Acid Substitutions in the Hexapeptide of Hoxa1 In Vivo Loss of Function but No Gain of Function Caused by Amino Acid Substitutions in the Hexapeptide of Hoxa1 In Vivo. Mol Cell Biol. 2004;24: 8567–8575. 10.1128/MCB.24.19.8567-8575.2004 15367676 PMC516739

[18] McNulty CL, Peres JN, Bardine N, van den Akker WMR, Durston AJ. Knockdown of the complete Hox paralogous group 1 leads to dramatic hindbrain and neural crest defects. Development. 2005;132: 2861–71. 10.1242/dev.01872 15930115

[19] Ruff JS, Saffarini RB, Ramoz LL, Morrison LC, Baker S, Laverty SM, et al Fitness assays reveal incomplete functional redundancy of the hoxa1 and hoxb1 paralogs of mice. Genetics. 2015;201: 727–736. 10.1534/genetics.115.178079 26447130 PMC4596679

[20] Meagher S, Penn DJ, Potts WK. Male-male competition magnifies inbreeding depression in wild house mice. Proc Natl Acad Sci U S A. 2000;97: 3324–3329. 10.1073/pnas.060284797 10716731 PMC16238

[21] Carroll LS, Meagher S, Morrison L, Penn DJ, Potts WK. Fitness effects of a selfish gene (the Mus t Complex) are revealed in an ecological context. Evolution. 2004;58: 1318–1328. 15266980 10.1111/j.0014-3820.2004.tb01710.x

[22] Ilmonen P, Penn DJ, Damjanovich K, Clarke J, Lamborn D, Morrison L, et al Experimental infection magnifies inbreeding depression in house mice. J Evol Biol. 2008;21: 834–41. 10.1111/j.1420-9101.2008.01510.x 18312317

[23] Ruff JS, Suchy AK, Hugentobler S a, Sosa MM, Schwartz BL, Morrison LC, et al Human-relevant levels of added sugar consumption increase female mortality and lower male fitness in mice. Nat Commun. 2013;4: 2245 10.1038/ncomms3245 23941916 PMC3775329

[24] Gaukler SM, Ruff JS, Galland T, Kandaris KA, Underwood TK, Liu NM, et al Low-dose paroxetine exposure causes lifetime declines in male mouse body weight, reproduction and competitive ability as measured by the novel organismal performance assay. Neurotoxicol Teratol. 2015;47: 46–53. 10.1016/j.ntt.2014.11.002 25446017 PMC4416947

[25] Ruff JS, Hugentobler SA, Suchy AK, Sosa MM, Tanner RE, Hite ME, et al Compared to Sucrose, Previous Consumption of Fructose and Glucose Monosaccharides Reduces Survival and Fitness of Female Mice. J Nutr. 2015;145: 434–441. 10.3945/jn.114.202531 25733457 PMC4336529

[26] Gaukler SM, Ruff JS, Galland T, Underwood TK, Kandaris KA, Liu NM, et al Quantification of cerivastatin toxicity supports organismal performance assays as an effective tool during pharmaceutical safety assessment. Evol Appl. 2016;9: 685–696. 10.1111/eva.12365 27247619 PMC4869410

[27] Acampora D, Gulisano M, Broccoli V, Simeone A. Otx genes in brain morphogenesis. Prog Neurobiol. 2001;64: 69–95. 11250063 10.1016/s0301-0082(00)00042-3

[28] Nelson AC, Cauceglia JW, Merkley SD, Youngson NA, Oler AJ, Nelson RJ, et al Reintroducing domesticated wild mice to sociality induces adaptive transgenerational effects on MUP expression. Proc Natl Acad Sci U S A. 2013;110: 19848–53. 10.1073/pnas.1310427110 24248373 PMC3856842

[29] Chalfin L, Dayan M, Levy DR, Austad SN, Miller R a, Iraqi F a, et al Mapping ecologically relevant social behaviours by gene knockout in wild mice. Nat Commun. 2014;5: 4569 10.1038/ncomms5569 25090970

[30] R Core Team. R: a language and environment for statistical computing. R Foundation for Statistical Computing Vienna, Austria; 2016 Available: https://www.r-project.org/

[31] Bates D, Maechler M, Bolker BM, Walker S. Fitting linear mixed-effects models using {lme4}. J Stat Softw. 2015;67: 1–48.

[32] Gavalas A, Studer M, Lumsden A, Rijli FM, Krumlauf R, Chambon P. Hoxa1 and Hoxb1 synergize in patterning the hindbrain, cranial nerves and second pharyngeal arch. Development. 1998;125: 1123–1136. 9463359 10.1242/dev.125.6.1123

[33] Studer M, Gavalas A, Marshall H, Ariza-McNaughton L, Rijli FM, Chambon P, et al Genetic interactions between Hoxa1 and Hoxb1 reveal new roles in regulation of early hindbrain patterning. Development. 1998;125: 1025–1036. 9463349 10.1242/dev.125.6.1025

[34] Makki N, Capecchi MR. Identification of novel Hoxa1 downstream targets regulating hindbrain, neural crest and inner ear development. Dev Biol. 2011;357: 295–304. 10.1016/j.ydbio.2011.06.042 21784065 PMC3176680

[35] Vitobello A, Ferretti E, Lampe X, Vilain N, Ducret S, Ori M, et al Hox and Pbx Factors Control Retinoic Acid Synthesis during Hindbrain Segmentation. Dev Cell. 2011;20: 469–482. 10.1016/j.devcel.2011.03.011 21497760 PMC3677862

[36] Erickson RP. Southwestern Athabaskan (Navajo and Apache) genetic diseases. Genet Med. 1999;1: 151–7. 10.1097/00125817-199905000-00007 11258351

[37] Holve S, Friedman B, Hoyme HE, Tarby TJ, Johnstone SJ, Erickson RP, et al Athabascan brainstem dysgenesis syndrome. Am J Med Genet. 2003;120A: 169–173. 10.1002/ajmg.a.20087 12833395

[38] Tischfield MA, Bosley TM, Salih MAM, Alorainy IA, Sener EC, Nester MJ, et al Homozygous HOXA1 mutations disrupt human brainstem, inner ear, cardiovascular and cognitive development. Nat Genet. 2005;37: 1035–1037. 10.1038/ng1636 16155570

[39] Bosley TM, Alorainy IA, Salih MA, Aldhalaan HM, Abu-Amero KK, Oystreck DT, et al The clinical spectrum of homozygous HOXA1 mutations. Am J Med Genet Part A. 2008;146: 1235–1240. 10.1002/ajmg.a.32262 PMC3517166 18412118

[40] Hansson B, Westerberg L. On the correlation between heterozygocity and fitness in natural populations. Mol Ecol. 2002;11: 2467–2474. 12453232 10.1046/j.1365-294x.2002.01644.x

[41] West SA. Sex allocation Princeton: Princeton University Press; 2009.

[42] Balenger SL, Zuk M. Testing the Hamilton-Zuk hypothesis: past, present, and future. Integr Comp Biol. 2014;54: 601–613. 10.1093/icb/icu059 24876194

[43] Shabalina SA, Yampolsky LY, Kondrashov AS. Rapid decline of fitness in panmictic populations of Drosophila melanogaster maintained under relaxed natural selection. Proc Natl Acad Sci U S A. 1997;94: 13034–13039. 9371795 10.1073/pnas.94.24.13034 PMC24258

[44] Bell G. Experimental genomics of fitness in yeast. Proc Biol Sci. 2010;277: 1459–67. 10.1098/rspb.2009.2099 20129976 PMC2871833

[45] Morran LT, Schmidt OG, Gelarden IA, Parrish RC, Lively CM. Running with the Red Queen: host-parasite coevolution selects for biparental sex. Science. 2011;333: 216–218. 10.1126/science.1206360 21737739 PMC3402160

[46] Carroll LS, Potts WK. Functional Genomics Requires Ecology. Adv Study Behav. 2006;36: 173–215.

