# Supplementary material for: Mouse fitness measures reveal incomplete functional redundancy of Hox paralogous group 1 proteins
Source: PLoS One. 2017 Apr 5;12(4):e0174975. doi: 10.1371/journal.pone.0174975 (PMC5381901; doi:10.1371/journal.pone.0174975)
Supplement: S1 Table — (PDF) [file pone.0174975.s001.pdf]

S1 Table. Summary of mixed model results for genotype frequencies in Hoxa1^{B1} treatment and control lineage heterozygous F_1 breeding cages.

***Hoxa1$^{B1(g)/+}$* × *Hoxa1$^{B1(g)/+}$* Homozygote Comparison**				
GLMM with Poisson distribution and logarithmic link (116 observations, 58 groups)				
Random effects	*Variance*	*Std. Deviation*		
Breeding cage (Intercept)	0.000	0.000		
Fixed effects	*Estimate*	*Std. Error*	*Z value*	*Pr(>|z|)*
Intercept	0.535	0.101	5.32	<0.0001***
Genotype *(Hoxa1$^{+/+}$)*	0.209	0.135	1.54	0.123
***Hoxa1$^{B1(g)/+}$* × *Hoxa1$^{B1(g)/+}$* Summed Homozygotes *vs.* Heterozygotes**				
GLMM with Poisson distribution and logarithmic link (116 observations, 58 groups)				
Random effects	*Variance*	*Std. Deviation*		
Breeding cage (Intercept)	0.000	0.000		
Fixed effects	*Estimate*	*Std. Error*	*Z value*	*Pr(>|z|)*
Intercept	1.338	0.067	19.89	<0.0001***
Genotype (Homozygote)	-0.042	0.096	-0.43	0.665
***Hoxa1$^{+(g)/+}$* × *Hoxa1$^{+(g)/+}$* Homozygote Comparison**				
GLMM with Poisson distribution and logarithmic link (170 observations, 85 groups)				
Random effects	*Variance*	*Std. Deviation*		
Breeding cage (Intercept)	0.000	0.000		
Fixed effects	*Estimate*	*Std. Error*	*Z value*	*Pr(>|z|)*
Intercept	0.681	0.077	8.83	<0.0001***
Genotype *(Hoxa1$^{+/+}$)*	-0.043	0.110	-0.386	0.700
***Hoxa1$^{+(g)/+}$* × *Hoxa1$^{+(g)/+}$* Summed Homozygotes *vs.* Heterozygotes**				
GLMM with Poisson distribution and logarithmic link (170 observations, 85 groups)				
Random effects	*Variance*	*Std. Deviation*		
Breeding cage (Intercept)	0.000	0.000		
Fixed effects	*Estimate*	*Std. Error*	*t value*	*Pr(>|z|)*
Intercept	1.268	0.058	22.03	<0.0001***
Genotype (Homozygote)	0.086	0.080	1.08	0.283

***Indicates a p value < 0.001.
