# Supplementary material for: Mouse fitness measures reveal incomplete functional redundancy of Hox paralogous group 1 proteins
Source: PLoS One. 2017 Apr 5;12(4):e0174975. doi: 10.1371/journal.pone.0174975 (PMC5381901; doi:10.1371/journal.pone.0174975)
Supplement: S2 Table — (PDF) [file pone.0174975.s002.pdf]

## S2 Table. Summary of mixed model results for allelic and genotypic counts of offspring born within OPAs.

| | | | | |
|---|---|---|---|---|
| **Reproduction Allelic Count** | | | | |
| GLMM with Poisson distribution and logarithmic link (Intercept at week 15, 30 obs, 3 groups) | | | | |
| *Random effects* | *Variance* | *Std. Deviation* | | |
| Population (Intercept) | 0.155 | 0.394 | | |
| Population (Slope) | 0.001 | 0.025 | | |
| *Fixed effects* | *Estimate* | *Std. Error* | *Z value* | *Pr(>\|z\|)* |
| Intercept | 3.887 | 0.104 | 37.32 | <0.0001*** |
| Allele (*Hoxa1*$^{+(g)}$) | 0.145 | 0.050 | 2.89 | 0.004** |
| Time | 0.031 | 0.015 | 1.99 | 0.046* |
| Allele (*Hoxa1*$^{+(g)}$) × Time | -0.010 | 0.007 | -1.38 | 0.168 |
| **Reproduction Homozygote Comparison** | | | | |
| GLMM with Poisson distribution and logarithmic link (Intercept at week 15, 30 obs, 3 groups) | | | | |
| *Random effects* | *Variance* | *Std. Deviation* | | |
| Population (Intercept) | 0.001 | 0.038 | | |
| *Fixed effects* | *Estimate* | *Std. Error* | *Z value* | *Pr(>\|z\|)* |
| Intercept | 2.503 | 0.074 | 33.62 | <0.0001*** |
| Genotype (*Hoxa1*$^{+(g)/+(g)}$) | 0.267 | 0.097 | 2.75 | 0.006** |
| Time | 0.029 | 0.024 | 1.18 | 0.238 |
| Genotype (*Hoxa1*$^{+(g)/+(g)}$) × Time | -0.017 | 0.013 | -1.26 | 0.207 |
| **Reproduction Heterozygote Comparison** | | | | |
| GLMM with Poisson distribution and logarithmic link (Intercept at week 15, 60 obs, 3 groups) | | | | |
| *Random effects* | *Variance* | *Std. Deviation* | | |
| Population (Intercept) | 0.018 | 0.133 | | |
| Population (Slope) | 0.001 | 0.031 | | |
| *Fixed effects* | *Estimate* | *Std. Error* | *Z value* | *Pr(>\|z\|)* |
| Intercept (Observed Heterozygotes) | 3.183 | 0.093 | 34.25 | <0.0001*** |
| Observed Summed Homozygotes | 0.159 | 0.071 | 2.24 | 0.025* |
| Expected Based on *Hoxa1*$^{B1(g)/B1(g)}$ | 0.015 | 0.073 | 0.20 | 0.843 |
| Expected Based on *Hoxa1*$^{+(g)/+(g)}$ | 0.282 | 0.069 | 4.09 | <0.0001*** |
| Time | 0.028 | 0.019 | 1.43 | 0.152 |
| Observed Summed Homozygotes × Time | -0.007 | 0.010 | -0.71 | 0.475 |
| Expected Based on *Hoxa1*$^{B1(g)/B1(g)}$ × Time | -0.015 | 0.010 | -1.51 | 0.131 |
| Expected Based on *Hoxa1*$^{+(g)/+(g)}$) × Time | 0.003 | 0.010 | 0.26 | 0.797 |

*Indicates a p value < 0.05,** < 0.01, *** < 0.001
