# Supplementary material for: Mouse fitness measures reveal incomplete functional redundancy of Hox paralogous group 1 proteins
Source: PLoS One. 2017 Apr 5;12(4):e0174975. doi: 10.1371/journal.pone.0174975 (PMC5381901; doi:10.1371/journal.pone.0174975)
Supplement: S3 Table — (PDF) [file pone.0174975.s003.pdf]

S3 Table. Summary of mixed model results for founder competitive ability within OPAs.

**Male Competitive Ability**				
GLMM with binomial distribution and logit link (Intercept at week 12.8, 70 obs, 3 groups)				
Random effects	*Variance*	*Std. Deviation*		
Population (Intercept)	0.023	0.152		
Fixed effects	*Estimate*	*Std. Error*	*Z value*	*Pr(>|z|)*
Intercept	-0.832	0.179	-4.64	<0.0001***
Genotype *(Hoxa1* $^{+(g)/+(g)}$*)*	0.411	0.210	1.96	0.050
Time	0.061	0.021	2.85	0.004 **
Genotype *(Hoxa1* $^{+(g)/+(g)}$) × Time	-0.090	0.029	-3.12	0.002 **

Indicates a p value < 0.01, * < 0.001
